# The War

**Published:** 1856-01-01

**Authors:** 


THE JOURNAL
OF
PSYCHOLOGICAL MEDICINE
AND
MENTAL PATHOLOGY.

JANUARY 1, 1856.

### Part First.

### ORIGINAL COMMUNICATIONS.

### Art. I.—THE WAR.

The moral as well as the physical world is full of the evidences of change, transition, and progressive development. Our public institutions and our language are but the fossil remains of a former state of society; just as the geologist shows us in certain strata the fauna and flora of species and genera long since extinct. The Latin tongue and the old Roman law are to be found in the statutes and language of every people in Christendom. Imbedded in present forms and manners, we pass them by without considering that mere testimonies of the past are as thick and frequent as the zoophytes that stud the limestone slab on which we are standing; that what is social is as antique as what is natural; and that we ourselves are but the children of a race subsisting amidst the repeated transmigrations of ages. It is the same with every other branch of science. Astronomers tell us that the fixed stars have a proper movement of their own; that new stars have appeared and old ones disappeared; that nebulæ are forming or have formed within the memory of man; and that aërolites are but the portions of a disrupted planet, circulating round the sun,

> "——— together hurl'd,
> The fragments of a former world."

The violent shocks of nature are not limited to our own globe, and meteoric, if not volcanic, agency is at work in the realms of space as powerfully as it is within the bowels of the earth.

If we descend from nature to man, the story is the same. They planted and builded, and married and were given in marriage, and the flood came and took them all away. Nineveh, Thebes, Babylon, Memphis, and Tyre are synonymous with war and miseries; and their names stand out like the salient angles of a bastion that conceals the cares of ordinary life behind its bristling parapets. The sculptured blocks recently arranged along the Museums of Paris and London, stammer forth, as voices from the dead, in broken hieroglyphics or arrow-headed inscriptions, the worn-out chronicles of their times. Nor do the arts and sciences of the present day change the burden of the song: steam and the electric wire only add velocity to life, and life, whether slow or fast, is evermore the same.

Upwards of a thousand years ago, the Greek Emperor, Theophilus, was, like ourselves, devoted to the progress of civilization.* He longed to surpass the splendours of Bagdad, at that time in the zenith of the caliphate. For this purpose, he engaged the most skilful artists to construct for him a toy of gold, set with precious stones, that played tunes of its own accord, like a musical snuff-box; and two golden lions that roared, and a golden tree with leaves rustling in the wind and boughs filled with singing birds. These puerile curiosities, together with many others of a similar kind, were enclosed within a spacious edifice, open to the admiration of all. It was the Crystal Palace of the ninth century—the harbinger of amity and peace. But, in humble imitation of our own, it was followed by war, a brilliant victory, a successful siege, abundance of spoils, and a triumphal return to the peaceful curiosities of the Crystal Palace. The war was renewed, but victory changed sides; and Theophilus died contemplating the bloody head of a rival held up by its hairs before his glazing eyeballs.

Suetonius tells us,† that when Augustus Cæsar heard of the loss of the three legions with their cohorts under Varus, he was inconsolable, paced his corridor, beat his head, and exclaimed in anguish, "Varus, give me back my legions!" But the legions were lost; and it was not till years afterwards that Germanicus fell upon their remains, and ascertained the precise spot of their destruction.‡ He traced the intrenchments, collected the bones of horses and men, and recognised some portions of armour mouldering into rust. When Tiberius was informed of this sentimental exploit, he was wroth, and rebuked Germanicus for leading the troops under his command to witness such dismal sights, and making them acquainted with so signal a reverse of the Roman arms. The rebuke was just; and though he was blamed

* "Histoire du Moyen Age." Paris: 1843. Tom. i. p. 524.

† Sueton. "in Oct. August." xxiii. "Per continuos menses, barba capilloque submisso, caput interdum foribus illideret, vociferans, 'Q. Vare, legiones redde;' diemque cladis quotannis mæstum habuerit ac lugubrem."

‡ Tacit. "Annal." i. 62. Quod Tiberio haud probatum.

for it at the time, Tiberius was in the right. For, in spite of his depravity, he was an astute politician, who, with exquisite discrimination, saw in this misfortune the earliest warnings of decay.

Xerxes, who was quite as luxurious, if not so debauched as Tiberius, was certainly just as farsighted in political affairs as that crafty old statesman. Herodotus gives us a peep into the Persian cabinet, and reports a speech of Xerxes upon the threatening rupture with Greece. "If *we* remain quiet," said the youthful tyrant, "*they* will not; for they will certainly invade us. Neither we nor they can stand still; we must attack them ere they attack us; they must submit to us, or we to them — there is no other alternative;"* and his pride instigated him to decide upon a war so plainly justified by his political acumen. But the decision cost him the severest trouble of mind. His sleep was broken, and his thoughts bewildered. In the night he was hallucinated; and Herodotus relates two of his spectral illusions. The war, however, was entered on; and the contest evoked the crisis that he dreaded. It hurried on Greece to its maturity; and not only were the Asiatics repulsed and chastised, but Persia, in her turn, trembled for her homesteads, her cities, and her fanes. Alexander came, and cut a high-road through the heart of her dominions.

The fifty years that followed the battles of Mycale and Platæa are the most brilliant and extraordinary on record; and we know the more about them, because their histories have reached us entire, and their well-known authors, Thucydides, Herodotus, and Xenophon were men of first-rate talents. In the midst of a rapid military career, by sea and land, the Athenians cultivated the liberal arts with the utmost enthusiasm, and carried them to a degree of perfection which few nations have been able to imitate, and none have surpassed. Under the administration of a single man, Athens was adorned with those magnificent structures, which Rome, on becoming the mistress of the world, did not disdain to avail herself of, in after ages, because she felt she was unable to copy what she could not but fall down and admire. So great, indeed, was the lustre shed by the name of Athens, that, from the age of Pericles, it designates the nation, country, and language of Greece. The Attic dialect, brought to perfection by its transcendent writers, became the model of all that is beautiful, and still holds the lofty privilege of being the most copious and the sweetest ever spoken by man.

The Persian war stands out in history as one of the landmarks of human greatness. The blood shed in battle was the first seed of liberty sown in Europe; but it required a series of wars—a succession of ages,—before it opened into blossom, and ripened

* Herod. *Pol.* xi.

into fruit; for the tree of liberty is no exotic of Eastern extraction. It is a hardy sapling that grows upon the bare ground or in the clefts of the rocks, luxuriates amid the ruins of empires, and strikes its roots the deeper the fiercer blows the blast.

The final cause of war is liberty. The immediate cause may be the gratification of personal ambition or national aggrandizement and pre-eminence; but its end is liberty. Man is not born free--he must fight for it. In all times and all places, slavery, in some form or other, has always been considered a necessary piece of state machinery. The moment absolute freedom is introduced, the state engine gets out of gear. This is the fact in republics as well as in monarchies and despotisms; for a republic is only a monarchy disguised. The smallest number governs the many, or a single genius rules them all; and the crowd follows its leader as blindly as sheep do their bell-wether. No ancient philosopher ever seriously entertained the notion of abolishing slavery and liberating two-thirds of his fellow-creatures. Plato and Aristotle were parasites afraid of offending the reigning fashion of the day, or at the best, they were only speculative sophists. Nor was it till Christianity had sapped the foundations of Paganism, that man awoke from his social lethargy, and found, to his amazement, that he was no longer a nonentity, either in time or eternity. Rousseau, in his "Social Contract," affectedly exclaims, *L'homme est né libre, et partout il est dans les fers!*—but Lucan more honestly makes Cæsar say, *Humanum paucis vivit genus:* * life is only for the few, and though freedom be our birthright, yet subjection or subjugation is the lot of most of us.

But to return to the moral or social cause of war. It is the effort for freedom. There is a moment in its affairs, when the world is stung to the quick with the sense of *Liberty*—a watchword that startles the repose of kings, breaks up the routine of life, and stuns the monotony of peace. Nations abhor foreign rule. The most respectable communities are those who govern themselves, or who are governed by their own princes, electorates, or presidents. This has always been the case. To be devoid of this nationality, is a proof that a people are already dead, or are ready to die. The foreigner is not only a conqueror, which is detestable, but also a tyrant, which is insupportable. His gauntlet is steel, and his sceptre iron. His dignity crushes those whom he pretends to govern, and the reaction is tremendous. There are some evils we cannot shun, but which must be faced, submitted to, or overcome; and rebellion or war provoked by tyranny is one of these.

Hence it happens that war, horrid as it may be, develops some of the noblest passions of the soul, and purifies the heart as much

* Lucan. "Pharsal."

as it enlarges the intellect. The condition of the weak, the needy, the ignoble, and the low is ameliorated by its means,—condignly, indeed, as each may suffer from it during its blood-stained progress. The penalty paid is enormous; every transition is a period of confusion and loss; nor is society ever revolutionized without its proportionate measure of suffering and woe. We are not its advocates—we are only stating the case.*

The next most eventful epoch in the history of mankind is the age of Julius Cæsar.† He was the most gifted man the world had ever seen, and his laurels are still green. As a warrior or a statesman, as an astronomer or a writer, as an architect or an orator, as a conqueror, a civilian, or an engineer, he stands alone and equally distinguished in each department. He is chiefly known as a great captain; but he was much more than a military man. Over the darkest portion of Europe he cast a gleam of light which has never been extinguished. His eye saw farther than the mere winning of a battle; for he was a diplomatist of the highest class, who reorganized those whom he subdued with the same masterly hand as that with which he had fought them in their own territories. By the firmness of his policy he delayed the fall of his country by the space of three hundred years or more; for he foresaw, not only what his political opponents

* "War, then," he said, "war, the grand impoverisher, is also a creator of the wealth which it wastes and devours?"

"Yes," replied Bridgenorth, "even as the sluice brings into action the sleeping waters of the lake, which it finally drains. Necessity invents arts and discovers means; and what necessity is sterner than that of civil war? Therefore, even war is not in itself unmixed evil, being the creator of impulses and energies which could not otherwise have existed in society."

"Men should go to war, then," said Peveril, "that they may send their silver plate to the mint, and eat from pewter dishes and wooden platters?"

"Not so, my son," said Bridgenorth. Then checking himself as he observed the deep crimson in Julian's cheek and brow, he added, "I crave your pardon for such familiarity; but I meant not to limit what I said even now to such trifling consequences, although it may be something salutary to tear men from their pomps and luxuries, and teach those to be Romans who would otherwise be Sybarites. But I would say, that times of public danger, as they call into circulation the miser's hoard and the proud man's bullion, and so add to the circulating wealth of the country, do also call into action many a brave and noble spirit, which would otherwise lie torpid, give no example to the living, and bequeath no name to future ages. Society knows not, and cannot know, the mental treasures which slumber in her bosom, till necessity and opportunity call forth the statesman and the soldier from the shades of lowly life to the parts they are designed by Providence to perform, and the stations which nature had qualified them to hold. So rose Oliver—so rose Milton—so rose many another name which cannot be forgotten—even as the tempest summons forth and displays the address of the mariner."

"You speak," said Peveril, "as if national calamity might be, in some sort, an advantage."

"And if it were not so," replied Bridgenorth, "it had not existed in this state of trial, where all temporal evil is alleviated by something good in its progress or result, and where all that is good is close coupled with that which is in itself evil."—WALTER SCOTT.

† Sueton. "in J. Cæsar," *passim.*

imputed to him, the coming empire, but also the overthrow of that empire by the very barbarians whom he was engaged in defeating. And never was there a greater blunder perpetrated than in his assassination. His murderers could not supply his place: they were disunited, soon dispersed,—some committed suicide, others treachery, and each of them came to ruin. The boy Octavius gathered up the fragments of the state, and cemented them into a whole, to which the name of Cæsar gave importance and renown. Now, as the Median war, five hundred years previously, had sown the first seeds of liberty in Europe, so the wars of Cæsar called into existence the first germs of civilization in the West. It was the *punctum saliens* of the modern world.

The question has often been agitated, whether Rome would have fallen if Carthage had stood. A glance will show that the fall of Carthage was only a matter of popular rivalry. The dominant power of the day must be all in all, or else it is nothing. Rome saw the alternative, and acted upon it with her instinctive sagacity and promptitude. Carthage was razed; but with her fell nothing but a nation of shopkeepers. For merchants are only money-dealers, whose views are no larger than their coffers, and whose ideas of national prosperity do not extend beyond their balance-sheet at the end of the year, or a clever speculation in some remote corner of the globe. A tradesman can never be either a statesman or a soldier. In Hannibal there is nothing akin to Napoleon, Cæsar, or Alexander. *They* were more than soldiers, while *he* was only a general; and Carthage, the centre of commerce and wealth, had not advanced the world a step forwarder in its progress, but left it where she found it, in the government of others, instead of her own. She has not bequeathed to us a single writer of note, her language is forgotten, and her spirit, except for trade, has left not a trace behind.

On the contrary, Rome consolidated the world, and held it together for many a long century; her fall loosened the bond, and scattered the growing nations around her. It was a moral earthquake greater than any that had ever yet shaken the framework of society. It was accomplished with war and disaster. The effect was instantly manifest: Christianity dared to show herself, and many nations arose out of the one. The cross, sentiment, and devotion crept forth from beneath the statues of the ancient gods, whose worship was now defunct, for the world had changed its mind and its tastes. Charlemagne, breastplates, shields, and the lance in rest became the novel order of the day. It was a pantomime on a gigantic scale. Cæsar, Cicero, Pompey, and Mark Antony would have found themselves sadly out of place in company with Odoacer, Clovis, Pepin, and all their chivalry.

Quickly, another phantom stalked upon the stage; it was Mahomet with his crescent, turban, and scymitar—Mecca, Kaaba, and the Koran—his cruel disciples, and the Saracenic host. Battle, and murder, and crime were not only the usual, but also the religious precepts in vogue among them. Opposition of principles begat contention. It was no longer the stale design of conquering many kingdoms on purpose to convert them into one, but of fighting for faith, as the essence of life.* The knight in armour was a reckless enthusiast, but a great favourite for the time being. His plume, his helm, his polished hauberk, thigh-pieces, and leggins, his gauntlet, shield, and spear, with its fluttering pennon and quaint device, was the gew-gaw that once represented the mind of the age, but which now graces in effigy the mantelpiece or bracket of some well-furnished drawing-room. Nevertheless, the cause was a real one; the man within the iron mask was in downright earnest; the Saracen was wrong, and the Christian was right; and Charles Martel proved it to be so, at the battle of Tours.† From that hour, the Crescent withdrew its diminished head, and, except for Charles, the *hammer* of the North, except for his Norman horsemen and their heavy arms, except for the blood spilt and the numbers slain on that memorable battle-field, we might, for aught we can see to the contrary, have been Turks instead of Christians at this present hour, worshipping Mahomet instead of Christ, both in England and France.

The eighth century dawned upon the world a wilderness of war, want, ignorance, darkness, crime, and famine, both spiritual and corporeal. It was chaos returned. But from this seeming confusion arose, like a morning mist, the present states of Europe, in their youth and prime. The old governments were gone, the new were unsettled and inexperienced. The Franks had conquered Gaul and Germany. Beyond the Rhine, fresh populations were in continual ebullition, menacing those on the south, or menaced themselves by others on the north. The Lombards possessed Cisalpine Gaul. A Duke governed Rome under the title of an exarch—a figment of the past. The little city of Gaëta was the only mart of promise, with municipal laws and a militia of her own. Upon a rock, by the sea, she protected the plains of Garigliano, the orange trees, the aloes, the cactus, and the African vegetation that still adorns that coast. It was a trading community, while in the volcanic region, the Solfaterra, of Vesuvius, Sorrento and Amalfi, with the mariner's compass, revived the commercial spirit of the Phenicians.

* "Ex multis gentibus nationibusque, unum regnum populumque constituit." (Justin.)

† Gibbon, c. 52.

The terrified inhabitants of Padua, who had fled from Alaric, passed over to the Rialto, at the top of the Adriatic Gulf; and the others, who subsequently retired before Attila, perched themselves on the neighbouring isles, like so many sea-fowl. They founded Venice, and became the merchant princes in a cluster of palaces emerging from the sea.* The Doge of Venice was a great man in his day. Arsenals, docks, ships, and trade arose at his bidding; and in strength and importance the merchant-city played no mean part among the other cities of the earth.

The British isles were still uncivilized. Spain, rescued from the Visigoths, fell a prey to the implacable Moors, who extinguished the light of faith everywhere, except the spark that smouldered in the Asturias. The Greek Empire, mutilated and dismembered, retreated from the Danube, abandoned the garrisons stationed by Justinian upon its right bank, and stretched a feeble arm of authority as far as Istria, Dalmatia, and Mount Hæmus. Africa, that had been either Greek or Roman, Palestine with Syria, the seat of the Caliphate, and the whole of Asia as far as Cilicia, had been seized on by the Arabs; and Arabia itself extended from the Euphrates to the Mediterranean, and from the Indus to the Iaxartes. The North was darkened by a mob of nomadic savages, who, under various designations, wandered and prowled from the Caspian to the Euxine, and from the Euxine to the Baltic.

Such was the scene disclosed as the curtain rose after the fall of Rome. It looks like a solemn masquerade, so motley are the figures, and so diverse from all that had gone before. There is no kindred feeling between the Dictator Sylla and Luke Anafetta, the first of the Doges—there is not the slightest resemblance between Attila and Scipio Africanus, Quintus Curtius and Leo the Great. The form, the features, the costume, the speech, the manners, principles of action, hopes, fears, plans, thoughts—all are changed; myriads have perished in the direful struggle; blood has flowed in torrents, cities have been sacked and destroyed, plains and villages ravaged with fire and sword, the mighty have been lowered, and the low exalted—everything upset: and all for what?

The fifteen decisive battles, so often spoken of as the fifteen critical turning-points of the world, are far less pregnant in their grand results than the five successful sieges which occurred in the course of the two thousand years that intervened between 500 B.C. and A.D. 1454. The siege of Babylon liberated the Hebrews; the siege of Sardis dissipated the wealth of Crœsus; the siege of Jerusalem by Titus dispersed the Jews; the siege of Rome broke paganism to pieces, and the siege of Constantinople

* "Histoire du Moyen Age." Paris: 1843. Tom. i. p. 273.

by the Turks started the modern period. For sieges are so much the more important in their consequences than battles, as cities or fortresses are the strongholds of empire, and the concentrated focus from which emanate those political ideas that govern a nation, a set of nations, or the world. Thus, the world fell when Rome fell, and with the fall of Constantinople the middle ages passed away.

A note was sounded, long, loud, and clear, at the fall of Constantinople, that re-echoed along the remotest shores of earth—it was the invention of the art of printing, the circulation of free opinion, and the discovery of the New World. It was the clarion-note of freedom and intelligence. It was the death-note of spiritual darkness, feudalism, and prejudice. It was the signal that awoke the giant-genius from his sleep. The mail-clad warrior raised his vizor, and glared around him; the monk threw back his cowl, and looked astonishment; the stoled priest paused on the steps of the altar, and listened; the turbaned Turk brandished his sheathless scymitar, and counted the years of his Hegira.* Remember, thundered Cicero in the ears of Mark Antony; remember, wherever thou art, thou art still within the limits of the Roman dominions!† That threat could now no longer be repeated. Beyond the sparkling waves of the Atlantic, another land was lying, ready to receive the fugitive of oppression, the victim of persecution, and the child of adventure. New plains, new rivers, new foliage, new mountains, and new scenes, invited the curiosity and courted the imagination of the Old World, standing on its shores, and gazing impatiently on the sea—"the fresh, the blue, the ever free!" The first discoverers came back, and told them of cataracts larger than their own, volcanoes loftier than Vesuvius, oceans without a storm—the smooth

* "No sooner was Mahomet sovereign of the city than the duration of the Ottoman sway was predicted. It was to last 400 years. No prophecy is more explicit, has been more widely extended, or has raised greater expectation. From the White Sea to the Persian Gulf it has been the belief of millions. Its origin we know not; but, unlike most predictions, it has been equally received by those who feared and those who hoped its fulfilment. Greek, Russian, and Turk have alike accepted it. It has stimulated the ambition of the Czars; it has encouraged the obstinacy of the Rayahs; it has unnerved and depressed the Turks, made them more reckless of the future, and more selfish in the concerns of to-day. The Christian has never ceased to speak of Roumelia as his country, and St. Sophia as his church; the Mussulman has acquiesced, and often seeks to bury his dead on the Asiatic shore, that they may rest in peace in their own land: natural causes esteemed so likely to have inspired and to be tending to fulfil the prophecy, that even Gibbon—no ready believer—gives an ear to its revelations. 'Perhaps,' he says, 'the present generation may yet behold the accomplishment of the prediction—of a rare prediction, of which the style is unambiguous and the date unquestionable.'"—*The Times*, Oct. 24, 1855.

† "Ubicunque terrarum sunt, ibi est omne reipublicæ præsidium, vel potius ipsa respublica," (Cicero "in M. Ant.," Phil. ii. perorat.) By this taunt, Cicero provoked his own destruction. In the game of life, the same cards that we deal out to others are dealt back again to us at the next deal.

Pacific—constellations unusually bright, a tropical climate, and a virgin soil. And well might man rejoice! What a blessed epoch in the course of events! What a prospect of a new order of things in the dull round of human existence! Nor, after the lapse of more than three hundred years, has the beneficent illusion lost its charm, for the sight of the southern cross, the Magellanic clouds, the coal-sacks, and stars, with a portion of the Milky Way, unseen in northern latitudes, the sight has, in our days, been alone sufficient to draw the aged Humboldt from his fatherland, and fix our own countryman, Sir John Herschel, in a fit of scientific ecstasy, at the Cape of Good Hope. There are now no longer any more lands remaining to be discovered, while those that have been discovered are already becoming macadamized as fast as possible. Civilization expels nature, and the smoke of science dirties the skies. The ancients found gold in the golden Chersonese, or India; the gold found by Philip of Macedon in Thrace, or Thessaly, served to suborn the orators of Athens; and the Phenicians worked the silver mines of Spain so well, that the prows of their ships and their anchors were made of the precious metal. Australia and California must be worked bare of their rich supplies at last; and then we must follow the guesses of geologists, and look for it anew in Japan, Kamschatka, or the Crimea.*

What we have written seems but the fiction of a poet, and yet what is the poetry of life but a fiction as unsubstantial as it is real? "I would the gods had made thee poetical," says Motley in *As You Like It*; to which Audrey most discreetly answers, "I do not know what poetical is." We, who are wiser than either Audrey or Motley, declare it to be the very essence of life. To be poetical gives animation to all we say or do, and gilds the vacuity of our days. The "Bride of Abydos," the "Lay of the Last Minstrel," or the "Rape of the Lock," is worth a thousand philosophical treatises; it is the wine after dinner, the summer cloud in the morning of affairs. It is in stirring times, and in seasons of excitement, that song resounds the best: the National Anthem never comes home to the feelings with so much force as it does on the battle ground of a day of victory. It is to these moments that we owe the patriotic

* Speaking of Philip of Macedon, Justin says, "auraria in Thessalia, argenti metalla in Thracia occupat." (Lib. viii. c. 3.)

"L'Espagne fut longtemps le Pérou de l'ancien monde. Près de Castalon, une montagne de la chaîne de la Sierra Seruga avait reçut le nom de Montagne d'Argent. Aristote rapporte que quand les Phéniciens débarquèrent pour la première fois en Espagne, ils firent une telle provision de ce métal, qu'au retour ils en fabriquèrent tous leurs ustensiles, jusqu'aux ancres de leurs vaisseaux." "Histoire Ancienne." Paris: 1845. Tom. i. p. 268.

Of the gold in Spain and Portugal, Justin says it was so abundant, "adeo etiam aratro frequenter glebas aureas exscindunt." (Lib. xli v. 3.)

airs of all nations: the Marseillaise Hymn, Partant pour la Syrie, the Croatian March, and God Save the Queen. The Scotch melodies in memory of Charles the Pretender originated in similar circumstances; and Moore's Melodies, although most of the airs may be traced to chants and litanies, arranged to his measure, or sometimes traduced to a jig, derive their popularity from a feeling of national sentiment. War has given rise to some of the best music; and the military band has greater charms for the cultivated as well as the uncultivated ear than the maudlin concerts and the sing-song of domestic life. Homer sang of a siege in a style that no man ever sang before; and Mr. Russell, the admirable correspondent of the *Times*, may, some thousand years hence, share with the Greek bard the safe and enviable honours of a poetic campaign. They both of them tell of things addressed to the common feelings of mankind, and music alone is wanted to render their records the most enchanting of their kind.

The love of glory is another passion inherent in the breast. It is not easy to define it. To love danger, and to delight in peril and woe, is a contradiction in terms; but then it is the pleasure of peril past and of danger overcome. But perhaps there is a deeper philosophy in it than this—namely, a profound sentiment of immortality. Wellington is reported to have said, "What will they say of us in England, if we lose this battle?"—the last he fought. It is evident the feeling was worth a bullet through the heart at the moment, or else it was worth nothing at all. To live after we are dead is a universal, but not a vulgar, passion. What is man without immortality? Nothing worth the name of man: a dead dog would do as well. To live hereafter is the hope of a rational being; to live in the memory of those we love is all that most of us desire; but to live in the everlasting regards of a nation of our own that we have served, is the highest ambition, "that last infirmity of noble minds;" there is nothing but heaven beyond it. War alone affords scope to the indulgence of a virtue so legitimately sublime as this. Yet they who die for earthly glory, know not the bauble for which they bleed. Democritus laughed, and Heraclitus wept, at the miseries of life, which point the shaft of satire with irresistible keenness. The celebrity of a great name is mostly consigned to the morning state of an army in the field, the obituary or casual remarks of a newspaper, the sealed registers at the Horse Guards or the Admiralty, the short-lived wonder of the town, or, at its best and rarest lot, the brief memory of a long-tried and solitary friend. Posthumous fame is the most precarious of commodities: the Wellington Despatches will live as long as the English language, and—so will Johnny Gilpin!

Besides glory and poetry, there are some meaner sentiments that would remain hidden in their own obscurity, except for the stimulus imparted to them by the parade and circumstance of war. Dress is one of these. Modifications of costume are referable to periods of violent convulsions. Weapons, offensive and defensive, are changed, and dress also: the breastplate gave place to the jacket, whether of cloth or leather, as the jacket is now yielding to the tunic; and the Greek fire, that foiled the lance and shield, gave way in its turn to the use of gunpowder. Satin and velvet belong to the piping times of peace, and the amorous moodings of a lady's chamber; but at the sound of the trumpet all is changed, from the fashion of the baldrick to that of the beard and headgear. For warfare is a great reformer, tantamount to the experience of a whole life, or of many lives in one. What is trifling flies away, and the useful, the grand, and the durable alone remains. Hence it comes to pass, that at the termination of great wars the chief actors involuntarily fall into the attitudes of the early drama, and the last act closes on a group exhibited in the happiest combination of colour, light, and shade. The spectacle is bright and evanescent: it passes away, like everything else; but the impression abides, and for a season the aspect of the world is changed.

Again, the weak and sickly die off. The old are removed by care or disaster. The young are cut short in their prime; none but the robust and vigorous survive the hardships of the times. The next generation increases in strength and stature; marriage is more frequent. Property, which had been tied up, is unexpectedly set free by premature or sudden deaths; and the destitute are supplied with fortune, while the young, who would otherwise have grown old in filial obedience and single blessedness, are placed in a position to gratify those longings, which all feel, but which so many are forced to repress from prudential motives. It is, so to speak, the harvest of the world, when multitudes are cut down and gathered to their account, and the field of life is gleaned to the last blade of mortality. What follows is a new epoch: the old are gone and the young begin a new career.

There is likewise an intermingling of nations; nation blends with nation, foreigner with foreigner. Antipathies clash with antipathies; hatred faces hatred; indifference confronts indifference. Nothing is idle; everything is on the alert. We test our capabilities and our reason; we measure swords, and measure things; discover that others are wiser than ourselves, and ourselves wiser or weaker than others. The divisions of province and country are levelled, and we perceive with surprise that those who dress in straw hats on the south side of that range of mountains are much the same as those in beaver on the north. This social fact staggers us just as much as the point of the bayonet in the deadly charge. Sometimes the straw hat conquers the beaver, and at other times the beaver the straw. We compare notes, settle differences, make concessions, compromise a great deal, embrace each other, and are friends. National prejudices are dissolved or softened; and we engage ourselves to observe the ordinary rules of fellowship for the rest of our days.

Language undergoes a change. The Assyrian superseded the Hebrew, and the Egyptian modified them both; the Greek swallowed them up, and the Latin swallowed up the Greek. Then came the modern tongues and dialects, each of them resulting from national discords, peculiarities, and contests. Nothing was done in peace; everything was done with strife. New ideas required new words, new phrases, and new technicalities. New knowledge creates a language of its own. The whole was metamorphosed, so that Pliny could not understand those who in our modern universities and colleges examine and lecture in Latin according to the rules of a Latin grammar unknown to the Latins of old. This farce is a shred of feudalism, and the sooner it is got rid of the better.

The Teutonic races, the descendants of Japhet, have hitherto conquered the world; and there seems no reason to suppose they will not continue to do so in future. They govern Europe, America, India, Australia, New Zealand, and Oceania—the northern and the southern hemispheres. What they have once acquired, they have always retained; and they will eventually conquer the entire globe, unless time should be no more, or they themselves should belie their name and origin. Their language superseded the Latin as early as the seventh century (A.D. 675). They may fail here or there, be defeated in this battle or lose that campaign, but the motto that waves from their masthead or flagstaff are the invincible words, *We will succeed*:—where there is the will, there is the way;

> "let both worlds rack,

> At least we'll die with harness on our back."

Who can withstand this? It challenges the world. Their ships navigate every sea, and their writings uphold the cause of freedom in every quarter of the globe. The Hanseatic League was theirs; theirs is the light of battle, popular elections, and free trade.

In peace, we may regulate our lives as we please; and, provided we keep within the pale of the law, and take care not to insult the conventions of society, we may indulge in luxury and vice to our hearts' contentment or bitterness. The sun shines, the air is tranquil, and all is still. We bask in the noontide warmth, and prolong our days in a sort of soothing dream. But it is not thus in war. In the clash of arms and the stern vigil of the foughten field, a dreary grandeur imposes both sobriety and caution. We cannot then mistake facts for fancies, nor realities for whims. There is nothing imaginative in a round shot, a shell, or a Minié ball—a wet tent, scanty rations, the dismal trenches, or an outlying picket. Nor is this forlorn feeling peculiar to the soldier on active service, for all feel it alike. The instability of affairs comes home to all. The rapidity with which everything is precipitated from life to death, and from certainty to uncertainty, forces the stoutest heart to quail, to meditate, and to reflect; and the miseries incidental to open hostilities throw a sable mantle of grief over many a tender soul far removed from the actual scene of violence and bloodshed.

Of the 25,000 or 30,000 soldiers who quitted these shores, full of health and spirits, in the spring of 1854, how many are now left alive to tell the tale? Only a few, a very few. Of the stalwart Guards and Highlanders, the complete regiments of the line, the well-equipped artillery, and the splendid cavalry, almost all are gone—even their gallant leader is no more; and well-trained horses without number, and men who were but as yesterday both veterans and heroes, are now numbered with the dead, the food of vultures, or dogs, or worms. The malaria of Gallipoli and Varna, the cholera, the glorious battles of Alma, Balaclava, and Inkermann, the weary siege, the storms of autumn, the winter's snow and frost, the damps of returning spring, tattered clothing, green coffee, starvation, did their worst, and changed them to

> "Such things—a mother had not known her son
> Amidst the skeletons of that gaunt crew." *

The matchless naval brigade suffered comparatively less than the troops, for the simple reason that they were near their own supplies, and Jack knows how to look after himself; although in the trenches they shared equal honours and equal dangers with the rest of the army. For the same reason, the Highland brigade and the marines fared better in the winter than if they had been posted well up in the front; but then these noble and unrivalled regiments preserved Balaclava for us:—*ils ne reculent jamais!*

"In times to come it will be a chosen terminus of Saxon pilgrimage, this Cathcart's-hill. Whether the traveller beholds from its humble parapet the fair aspect of the Imperial city, guarded by threefold mightier batteries than

* "Famine, despair, cold, thirst, and heat had done
Their work on them by turns, and thinn'd them to
Such things—a mother had not known her son
Amidst the skeletons of that gaunt crew;
By night chill'd, by day scorch'd, thus one by one
They perished." BYRON.

now, or sits upon the broken wall to gaze upon the ruins of Sebastopol, he must, if he has any British blood in his veins, regard with emotion that little spot which encloses all that was mortal of some of the noblest soldiers who ever sprang from our warrior race. He will see the site of those tedious trenches where the strong man waxed weak day after day and the sanguine became hopeless, and where the British soldier fought through a terrible winter with privation, cold, frost, snow, and rain, more terrible and deadly than the fire of the enemy. With the Redan, the Malakhoff, the Quarries, the Mamelon, Gordon's Attack, Chapman's Attack, under his eyes, he will revive with the aspect of the places where they stood the memories of this great struggle, and renew the incidents of its history. How many more of our gallant officers this cemetery may hold it is impossible to say; it is too full already. It is a parallelogram of about forty yards long by thirty yards broad, formed by the base of a ruined wall, which might in former days have marked the lines of a Tartar fort, or have been the first Russian redoubt to watch over the infancy of Sebastopol. Although many a humble tumulus indicates to the eye of affection the place where some beloved comrade rests till the last *reveil*, the care and love of friends here and at home have left memorials in solid stone of most of those whose remains are resting here."—*The Times*' Special Correspondent, October 8, 1855.

The sadness of heart inflicted by war is the penalty of sin or the inevitable condition of humanity. We must all die in our turn, but the anguish of parting for ever on this side the grave is the more poignant when the blow of separation is struck by the hand of man, instead of the more gentle operations of nature and decay. "Killed in action" means a violent, a sudden, or a cruel death: the consolations of a calm deathbed, surrounded by friends, a wife, a mother, a sister, or a child, are irretrievably wanting; the last words and the last kiss are given to the gory ground, and the last pressure of the hand is spent in struggling with the desperate foeman or grasping the weltering blade of slaughter. A bullet may dismiss the soul in a moment, or a heavier missile destroy vitality by a stun, at a long range, far off from the immediate conflict, but the end is the same—it is what the epitaph says, *Killed in action.*

Among the mental phenomena produced by war, the love of bloodshed is one of the most singular and revolting. It is not the savage villain, hackneyed in brutal deeds, that is alone prone to this horrible propensity; for, when once engaged in it, the gentle and the polite, the amiable and the mild, the handsome and the winning, become unconsciously infuriated with the sense of this diabolical thirstiness. When it is first spilt, and lies in a clotted pool upon the earth—the reeking earth!—the sight of the fresh blood inspires the awful passion. It is a mixture of fury and alarm, of ardour and revenge; nor can we accurately distinguish between bravery and a love of slaying—between the cold-blooded murderer whose deed is criminal and the bold soldier whose bloody work is glorious, patriotic, and praiseworthy. The motive alone excuses the deed, and the consent of mankind justifies the end. While it lasts, the passion is incontrollable; and, considered in its elemental form, it is clearly a madness.*

From this violent emotion arises another, which is akin to theft—it is the love for destruction and plunder. Those, who in ordinary life never think of appropriating what is not their own, nor of injuring, much less destroying anything good they happen to meet with, are instigated, in common with the rest of their companions, to demolish the fairest works of art, to ransack whatever is sacred and secluded, and to take possession of what does not belong to them, without the slightest hesitation and scruple. It belonged to the enemy, and it is theirs by the right of the war. Hence, the dreadful accounts we read of in the sacking, and burning, and overthrow of captured cities; as, for instance, that of Kertch in May, and that of Sebastopol, as far as its ruins allowed it to be so, in September last.

Connected with these two barbarous and debased propensities, let slip as the dogs of war, to torment man, is the excitement of the sexual passion:—but, enough! drop the veil over this lurid glimpse of hell, and bewail our fallen nature, and the victims of havoc and lust! We question whether any of those who have passed through these terrible ordeals, and have afterwards grown grey in the lap of peace, ever feel remorse or compunction arise within their breasts at the recollection of the share they have taken in the scenes they have witnessed? If not, it is an act of oblivion on the part of the moral sense, the more remarkable, because it occurs among Christian and civilized communities as frequently and as intensely as ever it did in the pagan and rude populations of antiquity; and it deserves the special attention of psychologists, moralists, statesmen, and divines.

Add to these vices, the habits of roving, and wandering, and restlessness, which are so contrary to domesticity, and which are acquired by soldiers and sailors from their particular modes of livelihood. A large army, accustomed to the field, or, in plain language, a select band of practised marauders, sent home and returned into store upon the conclusion of a peace, is the most grievous burden that can be laid upon an industrious people. They are so many consuming mouths which cannot supply themselves; and the ways of peace are no longer familiar to

* An anecdote is told of a young officer, of a remarkably mild disposition, who was engaged in the cavalry charge at Waterloo. He was accosted, on coming out of the affray for a moment, and asked why he wiped his bloody sword with so much eagerness? "We are here to kill our enemies," he replied; "and he is the best man who kills the most." With these words, he turned round and spurred his horse into the midst of the fight once more. Tacitus frequently mentions the gratification with which the legions *fleshed* themselves in slaughter. The idea is too shocking to be dwelt on.

them. But it is not thus with the sailor; for he can find his proper home again upon the waters, and his love of roving may be successfully turned in pursuit of trade and adventure, so conducive to the wealth, the happiness, and the greatness of a nation.

The last passion peculiar to warfare, is the state of mind engendered before, during, and after an action.* On the eve and morning of pitched battles, a sullen gloom pervades the camp—the silent presentiment of the coming event; which, as Banquo said on the night of his own murder, was a heavy summons, that lay like lead upon him. Officers and men are irritable and morose,—they are bending their courage to the point. At the first onset, the boldest are uneasy and reluctant, or rash and precipitate; but, when once in the midst of the engagement, the sense of danger is lost, delirium of a not unpleasing kind overwhelms them, together with a sensation, in some cases, of being lifted from the ground, and carried on by a preternatural movement. It is evidently hyperæmia of the brain, which subsides the moment blood flows from a wound, or the battle ceases; and then the paroxysm ends in fainting, or a disposition to shed tears, or apathy, or sleep.

Considering the subject *pathologically*, actual fighting appears in the light of an abnormal condition of the *cerebro-spinal system;* and were we, as pathologists, to assign to it its proper place in our nosology, it would be among the Protéan forms of exalted nervous sensibility. But we dare not affirm that an entire community, or several communities at once, should thus transgress the bounds of reason and discretion, when we see the greatest minds deliberately engaged in conducting the greatest wars to a prosperous termination. There is no doubt, war occasionally assumes an epidemic character, the result of a morbid principle of imitation—like suicide, and some of the convulsive diseases; while, among populations of an excitable temperament, this propensity is so easily called into action, that the slightest provocation is enough to kindle the spark, and light up the flame of revolution, civil contest, or foreign invasion, almost without the pretext of a *casus belli.* The lively people of ancient Greece might furnish many an instance in proof of this. It has likewise been remarked, that periods of warfare are usually associated with seasons of rare meteorological phenomena,—such as earthquakes, tempests, droughts, volcanic eruptions, comets, bad harvests, and epidemic mortality; so that the phrase of

* Tacitus, describing the feelings that pervaded a camp on the night previous to a battle, says:—"invalidi ignes, interruptæ voces, atquæ ipsi passim adjacerent vallo, oberrarent tentoriis, insomnes magis quam pervigiles. Ducemque terruit dira quies," &c. ("Annales," i. 65.) The madness of victory is proverbial.

war, pestilence, and famine, is both historically and scripturally correct. These phenomena, moral and natural, taken together, impart a formidable character to the passion for bloodshed, of which the preceding account is but its natural history, as it is exhibited in the histories of nations.*

It is the autumn of life, and the storms are stripping the leaves for the ensuing winter of the world. The migratory birds are on the wing; their time is short, and they are taking to flight or ever the frost and the snow stamp their cold seal on the hybernating death of nature.

> "Like the leaves of the trees when the summer is green,
> That host with their banners at sunset was seen;
> Like the leaves of the trees when the autumn is blown,
> That host in the morning lay withered and strown.
>
> "There lay the steed with his nostril all wide,
> But thro' it there rolled not the breath of his pride;
> And the foam of his gasping lay white on the turf,
> And cold as the spray of the rock-beating surf.
>
> "And there lay the rider distorted and pale,
> With the dew on his brow, and the rust on his mail," &c.
>
> BYRON.

Were finer lines than these ever penned by poet?

Never was a siege undertaken on a soil more replete with classical legend and historic recollections than that of Sebastopol. In the Crimea, the Tauric Chersonesus of the ancients, upon the borders of the Black Sea, the Pontus Euxinus of the Latins and Greeks, beyond the Thracian Bosphorus, and close to the Palus Mæotis, or Sea of Azoff, the moderns have played a memorable part in the annals of modern warfare. It was of this

* "On the field of battle one soldier at the appearance of blood experiences the intoxication of carnage; another will swoon at the same sight. Sir Walter Scott, in the poem in which he has referred to the battle of Bannockburn, alludes to the various feelings that influence the mind in the heat of an engagement; and, it will be perceived, that he directs particular attention to those who are influenced by no other motive than the pleasure they derive from sacrificing human life:—

> "'But oh! amid that waste of life,
> What various motives fired the strife!
> The aspiring noble *bled for fame,*
> The patriot for his country's claim;
> This knight his youthful strength to prove,
> And that to earn his lady's love;
> *Some fought for ruffian thirst of blood;*
> From habit some, or hardihood;
> But ruffian stern and soldier good,
> The noble and the slave,
> From various cause the same wild road
> On the same bloody morning trode
> To that dark inn, the Grave.'"

The "Anatomy of Suicide." By Forbes Winslow, M.D.

spot that Euripides chanted his real or fabulous tale of "Iphigenia;" and there it was that the Greeks actually reclaimed the Tauri from their brutal manners. There they fixed their maritime stations, perhaps in Balaklava itself, or the now half-calcined harbour of what was once Sebastopol. There, where the Cimmerian Bosphorus joins the Euxine with the Mæotis, is situated Kertch, or Panticapæum, the site of Cæsar's far-famed *bon mot*, or pithy despatch, of *Veni, Vidi, Vici.* The Romans once advanced within three days' march of the Tanais, or Don, the native ground of the marauding Cossacks, and the boundary between Asia and Europe. In frail flat-bottomed barks, framed of timber only, without a particle of iron, and covered with nothing but a slender roofing of reeds, the Goths carelessly trusted themselves to the mercies of an unknown sea. Their natural daring, and the hope of plunder, stimulated their ignorance and inexperience. They pillaged the Crimea; but at a later period the republic of Kherson assisted Constantine against them. The Genoese, the Venetians, and the gallant Franks, in their turn, penetrated those distant waters in pursuit of gain or adventures, till at last they fell under the rule of the Tartars, the Turks, and the Russians.

The basin of the Black Sea is a volcanic hollow, looked into by the snowy Caucasus, the heights of Ararat, and the shores of Mithridatic Pontus. Sinope furnished a god to Egypt, and the delta or liman of the Danube was the pitiful spot of Ovid's exile, for what he had seen—*quod vidi*—in the halls of Augustus Cæsar.* Some of the largest rivers of Europe empty their floods into its stormy bosom, which is as black as it is fathomless:† and an old proverb declared the mariner to be a fool who entered the Euxine before the ides of May, or tarried in it after the kalends of October. Yet such is the sea navigated by our ships of war, which have floated upon it throughout the whole year, prepared for action, and scatheless of shipwreck or disaster.

The long line of the Danube, from Galatz at its embouchure

* "Cur aliquid vidi?—cur noxia lumina feci? (Ovid. "de Ponto," lib. ii.) He says "Carmen et error" was the cause of his exile: the verses were Ovid's own, but the error is the supposed incest of Augustus with his own daughter. For such a sight, if true, banishment was only less than death. He makes the same allusion in other places.

† A young friend of ours, who has lately joined his regiment in the Crimea, says, speaking of his voyage thither: "The water of the Black Sea is certainly black. I was very much struck with its dark appearance on our passage from the Bosphorus to Balaklava."

"It is very deep, no bottom having been reached with a line of 140 fathoms."—Mrs. Somerville, "Phys. Geogr." 1851. Vol. i. p. 359.

Upwards of forty rivers, many of them the largest in Europe, flow into it. It receives the melted snows from the Caucasus, Ararat, the Balkan, the Carpathian and the Swiss mountains; and the waste waters from Central Russia and the Oural mountains.

up to Singidunum or Belgrade, on the Austrian frontier, is overcharged with mediæval and imperial recollections; Trajan's Wall at the Dobrudscha and Hadrian's Bridge beyond Kalafat are too well known for more than a casual allusion to them; and it was between these two extremities that the barbarians rushed across in winter, when the river was frozen over. The most active of the Roman emperors were continually engaged in fortifying this long extent of open country against their frequent incursions: here Aurelius earned or lost his laurels, and so did Probus and others; here were signed the famous treaties of Unkiar, Skelessi and Balta Liman; and here did Omar Pasha win his undying military renown. It was here that the Goths crossed over in force in the fourth century, seized upon the present point of Schumla, passed the defiles of the Balkan, descended into Roumelia, and fought and defeated the Emperor Valens near Adrianople. Valens,* with his personal staff, took refuge in a cottage, which the Goths set fire to, and burnt both him and his officers to death. It was in Gallipoli that the Turks first set foot in Europe. The whole locality is full of the living past: Mount Olympus, Achilles, and Troy—the Cyanean rocks, or floating islands, Hero and Leander, and the Argonauts.

The modern and ancient worlds have touched each other. The difference of mind and manners is immense. Steam brings us in communication with the land of Cimmerian darkness in ten days over a distance of three thousand miles, and the electric telegraph in as many hours. The mode of warfare is also frightfully different. The Russians lost 200,000 men in less than a twelvemonth, and the Allies can have lost scarcely less. Perhaps the grand total of 400,000, both sides taken together, is not too large a number to put down to the score of battle and disease in the short space of time that elapsed between the siege of Silistria and that of Sebastopol. The defence of Sebastopol alone cost the Russians, according to Prince Gortschakoff's account, "from 600 to 1000 men a-day for the last thirty days;"† and when the Allies triumphantly entered the fortress, they found the dead piled up in the streets, and mutilated limbs stowed away

* Gibbon, c. xxvi.

† "No English writer would have dared to rate the Russian loss so high; it would have appeared an ignorant exaggeration. 'During thirty days,' says Gortschakoff, 'the garrison lost from 600 to 1000 men a-day.' This is independent of the slaughter of the last three days' bombardment, and in the last supreme struggle during six terrible assaults. What the Russian losses have been during the whole campaign it is scarcely possible to conjecture, for we believe that no adequate idea has been formed of the terrors of this wonderful siege. Disease, cold, and combat have laid men low in numbers which it requires some boldness to state. We say nothing of the allied loss, but that of the Russians seems likely to have fallen little short of 200,000. Never in modern times has there been so great a destruction on so limited a field."—*The Times*, Oct. 8, 1855.

in empty barrels. We turn from the account with sickening horror; yet the necessity was stern and unrelenting. Russia was stealing a march on the south of Europe: had she conquered Turkey, she would have made a flank movement on Italy, Austria, and Spain; had she not been checked, France must at last have fought her on her own borders, and we along our own shores. The necessity was obvious; and the people, with their natural sagacity, perceived the dilemma, and boldly extricated themselves from between its horns by insisting on war.

The present contest will strengthen more than ever the cause of freedom and the power of the people, who prove themselves to be as far-sighted in *their* diplomacy as the most finished diplomatist ever pretends to be in *his*. The result is already visible in the temperate but firm tone with which the British nation continues to address itself to the war, and bear the necessary cost of its being carried on to a successful issue. The liberality with which they provided for the sick and wounded was not less estimable than unpretending. They openly did their duty, without looking for applause or recompence. A frame of mind of this cool and deliberate character is more than an omen of success, because it is the means of success itself, and the inferences to be drawn from it in favour of the future are bright and encouraging. The first Napoleon said, "Fifty years after my decease, Europe must make up its mind to become Republican or Cossack." That crisis has arrived, and France and England united have faced the rising foe on his own ground, and encountered him in the secret lair of his vast dominions.

The end of war is peace, and peace upon a higher elevation than it was before the commencement of the struggle. Examples without end could be adduced from history to prove that right ultimately prevails over might, and that the poetic justice awarded in works of fiction, is but the conclusion drawn from our experience of the world; for were it otherwise, we should be disappointed, because its failure would not be in accordance with profane or sacred truth. But it is from this confidence in the course of events that we so fearlessly rely upon the fate of arms—a proceeding which, though it may be discountenanced by the consent of mankind, yet is practically found to be the surest, if not the only means left for determining the balance of power or equity in the final adjustment of affairs. War is, therefore, the trial by battle on a large scale, in which thousands die instead of one, and the magnitude of the question at stake involves the welfare of millions instead of the particular interests of a king, a noble, or a plebeian. Nor is the appeal to Heaven in vindication of ourselves one iota less sincere and legitimate than the appeal to our drawn sword; since the dangerous expedient of leaving the justice of our cause to the arbitrement of Heaven or of arms, seldom betrays us. The experiment is not likely to prove too much for itself—the capricious choice of victory decides in favour of the injured party. A single battle or campaign may apparently go against this superstitious dogma; but, in the long run, success protects the deserving, and war never fails to yield the triumph incontestably in favour of truth, of justice, and of peace.

---

